# Recent Progress and Approaches on Carbon-Free Energy from Water Splitting

**DOI:** 10.1007/s40820-019-0335-4

**Published:** 2019-11-22

**Authors:** Aslam Hossain, K. Sakthipandi, A. K. M. Atique Ullah, Sanjay Roy

**Affiliations:** 1 Department of Physical and Inorganic Chemistry, Institute of Natural Science and Mathematics, Ural Federal University, Yekaterinburg, Russia; 2 Department of Physics, Sethu Institute of Technology, Kariapatti, Tamil Nadu 626 115 India; 3 Nanoscience and Technology Research Laboratory, Atomic Energy Centre, Bangladesh Atomic Energy Commission, Dhaka, 1000 Bangladesh; 4 Department of Chemistry, Shibpur Dinobundhoo Institution (College), Howrah, West Bengal 711102 India

**Keywords:** Water splitting, Renewable energy sources, Hydrogen generation, Photocatalysis, Nanostructure

## Abstract

Sunlight is the most abundant renewable energy resource, providing the earth with enough power that is capable of taking care of all of humanity’s desires—a hundred times over. However, as it is at times diffuse and intermittent, it raises issues concerning how best to reap this energy and store it for times when the Sun is not shining. With increasing population in the world and modern economic development, there will be an additional increase in energy demand. Devices that use daylight to separate water into individual chemical elements may well be the answer to this issue, as water splitting produces an ideal fuel. If such devices that generate fuel were to become widely adopted, they must be low in cost, both for supplying and operation. Therefore, it is essential to research for cheap technologies for water ripping. This review summarizes the progress made toward such development, the open challenges existing, and the approaches undertaken to generate carbon-free energy through water splitting.

## Highlights

- Different approaches for efficient carbon-free energy from water splitting are summarized.
- Step-wise evolution of water splitting research is highlighted with current progress.
- It describes the open challenges of charge transport properties and future research direction.

## Graphical abstract

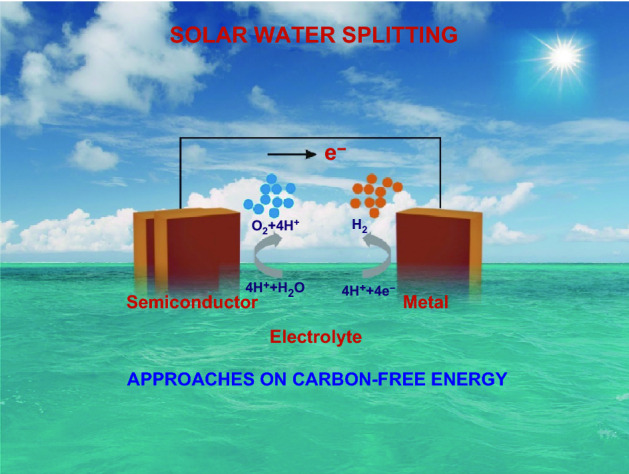

## Introduction

The world’s power supply relies predominantly on non-renewable energy resources such as oil, coal, and fossil fuel (natural gas). These geological resources have accumulated over countless millions of years through photosynthetic activity. Therefore, such fuel is representative of ‘solar energy’ [1–3]. However, steep rises in energy consumption over the last century have resulted in an inevitable shortage of those valuable resources. This impact is already being seen to a lesser extent with the economic, social, and political changes. Increase in human population and economic process, especially in fast-developing countries, can result in an additional increase in energy demand. Furthermore, the burning of carbon-rich fuels has redoubled the carbon dioxide concentration in the atmosphere. This increase is capable of influencing evolution and climate changes, with projected adverse effects for our planet and the human society [4]. Therefore, all countries need to challenge themselves to identify and develop various alternative energy sources [5, 6].

Solar power is by and far the most important source of renewable energy (~ 1.2 × 10^14^ kJ is received by the earth’s surface every second) [7].Therefore, it is only natural that there is significant interest to trap this irradiation and chemically apply it to generate energy [8]. Consequently, in recent years, there has been an outburst of research to target the direct solar energy for the production of fuel devices. Such direct solar-to-fuel devices may be considered to be ‘artificial photosynthesizers,’ as they capture the daylight energy, use it to react with chemical bonds, and produce a final product that is referred to as ‘solar fuel’ (in this case, H_2_) [9].

Currently, several review articles have reported on water splitting (WS) specifically. Numerous recent reports have dealt with WS research on the surface engineering of nanomaterials [10], plasmon-enhanced visible light-driven WS [11], surface and interface engineering for photoelectrochemical water oxidation [12], mechanistic understanding of the plasmonic enhancement for solar WS [13], and defects engineering in photocatalytic WS materials [14]. Therefore, this review aims to highlight the current progress, the approaches on carbon-free energy sources, and step-wise theoretical and experimental research on WS.

This review intends to summarize the recent evolution in the theoretical approaches for WS and the various cost-effective strategies developed. This is dealt in the context of the solar energy’s potential use in photoelectrochemical solar-to-H_2_ devices. We aim to reinforce the demand for whole-system approaches, especially among those who are already dealing with electrocatalytic WS. However, this review is especially meant to educate and encourage those who are new to this field and challenge them to contribute to this fascinating, ever-changing area of research. Information regarding in-depth strategies applied for electrocatalytic chemical element evolution (H_2_ evolution) [15] and gas evolution [16] (oxygen evolution) is dealt with in this review. We focus totally on the progression made by the various elementary studies in this field, in addition to short highlighters regarding the various recently developed approaches to get carbon-free energy by WS.

## Revolution of Water Splitting Research

Since the primary demonstrations of photoelectrochemical WS in the 1970s [17], several resources and analyses have been dedicated to develop affordable elements for generating solar fuel systems. Previous reviews surveyed the information regarding the physical and thermodynamic principles involved in H_2_ and O_2_ evolution reactions, the performance of assorted semiconductors, the configurations of photoelectrochemical cells, the properties of catalysts, and the influence of assorted structural influences on the efficiency of the method [18]. They additionally summarized the values obtained for the energy conversion efficiency or the alleged solar-to-atomic number 1 efficiency, which were obtained for WS cells of various configurations. The values varied between 0.01 and 18%, that is, by an element of concerning 2000, for photoelectrochemical cells while not surface catalysts and people with catalysts and buried electrical phenomenon junctions (photovoltaic).

To technically understand the process, direct solar-to-chemical energy conversion in photoelectrochemical cells is described, with emphasis on economical and cost-effective techniques for H_2_ production. The techniques were compared with the method that involved electrolysis of water for photovoltaic-generated electricity. It also deals with integrating light-weight energy usage and WS into a single device [19]. This goal, however, poses serious material-related challenges: Semiconductor photoelectrodes ought to expeditiously harvest solar irradiation and drive water oxidation or reduction reactions with a sufficient rate of current densities (10–15 mA cm^−2^) in liquid solutions underneath one Sun illumination while not degrading for an appropriately extended period of time, that is, over 2000 h consistent with benchmarks set by the Department of Energy, the USA [20]. All these aforementioned aspects cannot be met by a single material, and therefore, formation of assorted heterostructures [19] is not possible. Bicycle-built-for-two (tandem) devices [21] have been developed for photocorrosion protection of semiconductors [22]; however, they have not proven to be efficient in its usage during 2010–2016, as is seen by the documented literature.

The highest solar-to-H_2_ efficiencies that exceed 10% have been until now generated using costly and unstable semiconductors that belong to the III to V groups [23]. Therefore, the central focus of a research in this field is to discover economically viable, efficient, and stable material compositions for photoelectrochemical WS, especially among elements abundant on the earth. Most Si photovoltaic cells are being deployed to generate solar H_2_, as this technology is not only well developed already but is also additionally significant as the cost of the crystalline chemical element in the star cells has reduced over seven times within the past few years [24]. Despite the fact that there has been vital progress in understanding the physical challenges involved in the fabrication of sensible solar fuel generators, the analysis of the technoeconomical implications for their deployment has been limited. These value implications are extraordinarily necessary for realizing business implementations of solar fuel technologies.

**Fig. 1:**
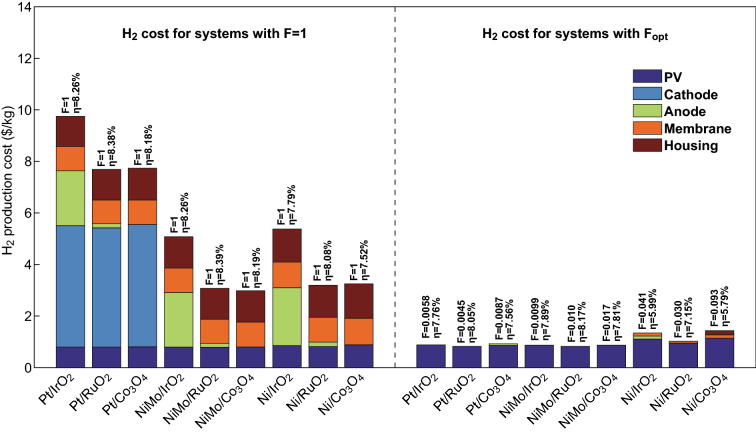
Cost comparison and evaluation of solar–H_2_ generators that integrate different catalytic components. Reproduced from Ref. **[**26**]** with permission from the Centre National de la Recherche Scientifique (CNRS) and The Royal Society of Chemistry

One of the major challenges for controlling the price of solar-H_2_ systems is related to the shortage in reference demonstrators for base calculations. Despite this challenge, many studies in the literature have provided insights in the value and energy needs for solar-H_2_ production using designs believed to be promising candidates [25]. In addition, a comprehensive sensitivity analysis on system parameters (both physical and economic factors) is under progress to elucidate their overall impact on the price of H_2_ created. This approach allows for a good comparison among the various solar–H_2_ generation systems based on cost-effectiveness and benefit ($/kg of H_2_). Rodriguez et al. [26] primarily outlined the price of H_2_ on the basis of the light-absorbing element (up to 97% of the cost), stating that the material choice for electrolysis has, to a large extent, minor effects. The findings discussed here will facilitate direct analysis and help develop deployable solar–H_2_ generators that have competitive value in relation to energy sources that may be efficient in terms of cost. Figure 1 shows the price (left graph) of H_2_ for all attainable combinations of catalysts with *F* (quantitative relation between electrolysis area and photovoltaic area) = 1 (i.e., corresponding to the magnitudes of solar fuel generators based on photoelectrodes) and (right graph) an optimized value for *F*.

Reece et al. [27] developed solar WS cells consisting of triple junction amorphous Si electric cell interfaced with cobalt borate and NiMoZn as O_2_ and H_2_ evolution catalysts, respectively. The best-obtained solar-to-H_2_ efficiency rate was 4.7% underneath one Sun illumination; however, a stable operation could be conducted on the device for just 10 h. Chen et al. [22] successfully stabilized chemical element photoanode by atomic-level layer deposition/coating of 2 nm thick using TiO_2_ lined with 3-nm-thick metallic element film; this was suitable for oxygen evolution by catalysis reactions. Stable operation of the anode was ascertained for a minimum of 24 h. Similarly, atomic layer deposition-grown Al-doped ZnO (20 nm) and TiO_2_ (20 nm) protective layers changed with Pt nanoparticles have been shown to effectively stabilize Cu_2_O photocathode [28] for H_2_ production, which retained 62% of the initial photocurrent value after 10 h stability check. Kenney et al. [29] set a record of 80-h-long direct water oxidation using n-type Si photoanodes passivized with a 2-nm-thick nickel film, which acted additionally as an oxygen evolution catalyst.

Considering the aforementioned research, the apparent ‘brute-force’ or photovoltaic reaction along with the electrolysis phenomenon is recently being reconsidered [30]. The benefit of this technical scheme is that both processes, that is, the photovoltaic and electrochemical energy conversions, can be optimized independently. Moreover, the issues connected with blocking of the photosensitive surface with H_2_ or O_2_ evolution reaction catalyst particles and photocorrosion of semiconductors are mechanically avoided. Recently, solar-to-H_2_ efficiency exceeding 10% was achieved with a noble metal, with crystalline silicon photovoltaics module and low-value H_2_ and O_2_ evolution reaction catalysts [25]. About 12.3% solar-to-H_2_ efficiency using inexpensive perovskite photovoltaics and earth-rich catalysts has been reported recently [30]. Although the results are promising, the instability of perovskite electric cell limits the time period of WS device. For efficient applications, the expected time period for a solar-powered device that is run by solar cells should be 25 years. Thus, it is evident that for construction of an economical solar WS system, a trade-off between potency, cost-value, and longevity should be achieved.

## Different Approach for Water Splitting Research

The current status of energy demand drastic conflict with the limited fossil fuel supply in nature which is inspiring to research for the development of sustainable energy sources. Solar energy is one of the flexible and universal promising candidates which can be utilized by photovoltaic and photothermal approaches. So, the conversion of solar energy to chemical energy is very important to solve the future energy problem. The several theoretical and experimental approaches for photoelectrochemical WS are important in modern science which is discussed here.

### Plasmonic Solar Approach for WS

Plasmonics is a promising functional field of science and technology, which exploits the unique optical properties of nanometallic structures to manipulate light and its pathways at nanometer length scales. Nanometallic objects develop their properties from the ability to maintain collective electron excitation, known as surface plasmons. Currently, plasmonics research has enabled new fundamental science and instrumentation technologies. Plasmonics may soon become a widespread technology, offering rare optical capabilities and the opportunity to achieve unprecedented intensity levels of synergy between optical and electronic functions. Although resistive heat loss in metals can severely limit the enactment of devices that rely critically on long-range propagation of waves on surface plasmon (surface plasmon polaritons), despite the presence of losses, recently, numerous useful functionalities have been realized. The most prominent ones are related to practice structures for excessive light concentration, such as lenses, resonators, and plasmonic antennas; all of these are excellent examples of this technology [31–33].

Plasmon enhancement is an exciting track route to control the drift of electromagnetic energy in photocatalytic and photoelectrochemical WS devices. Energy transfer during plasmon resonance enables precise spatial control over photon absorption in a semiconductor. This approach is particularly beneficial in materials where photocurrent is limited by a small minority carrier transport distance, although the full range of the advantages of plasmon resonance transfer energy has yet to be explored. Many emerging semiconductors, which are both of scientific and economic interest, such as hematite, are excellent candidates for studying the plasmonic effect because these similar or same semiconductors often have high defect densities that promote recombination.

Plasma resonance energy transfer can provide advantages for generating less expensive or rare semiconductors by reducing the amount of semiconductors required for photoabsorption. Through plasmonics, semiconductors may become by and large capable of absorbing the same quantity of light using third-order magnitude lower semiconductors [34], thereby effectively focusing light and increasing photovoltage and efficiency. It may also be possible to fabricate a device with a smaller semiconductor–electrolyte interfacial region than the projected area of the substrate, leading to a dramatic upturn in photovoltage.

Although metals have high inherent chemical activity and the ability to selectively activate many chemical transformational changes, it has been assumed that the percentage of photocatalytic reactions on metals at photon intensities such as that of the Sun is characterized by energetic charge carrier formation and short lifetimes. However, these are very few due to low capacity [35]. It was recently shown that unlike metal structures, plasmonic photoexcited nanostructures show relatively high photocatalytic activity when exposed to photons of Sun-like intensity [36]. So far, observations on plasmonic nanostructures of direct photocatalysis have been restricted to partial exothermic oxidation only, selective reduction, and decomposition organic chemical reactions on excited plasmonic Au and Ag nanostructures.

**Fig. 2:**
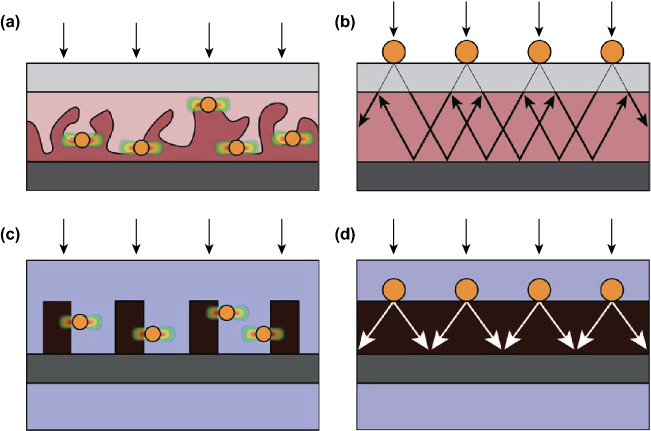
Device proposal/design for solar cells (**a**, **b**) and analogous designs for photoelectrochemical cells (**c**, **d**). Reprinted from Ref. **[**42**]** with permission from the Centre National de la Recherche Scientifique (CNRS) and The Royal Society of Chemistry

Organic solar cells (Fig. 2a) typically exploit metallic nanoparticles of small particles to lead to heterojunction where separation of charge occurs [37], although other geometries have also been explored [38]. This allows light to be localized at the interface between two phases. Inorganic solar cells (Fig. 2b), such as those established on silicon *p*–*n* junctions, have generally exploited plasmonic nanoparticles positioned at some distance from the *p*–*n* junctions. In most cases, the primary mode of operation is by light scattering [39], although some contribution of near-field effects is possible [40]. In WS devices, the electric field that separates electrons and holes is located at the semiconductor–water interface. Unlike most conventional inorganic solar cells, which have a buried space charge layer, metal nanoparticles can be placed at the semiconductor–water interface, allowing near-field energy transfer from semiconductor to metal in a manner consistent with organic solar cells. Additionally, scattering effects may additionally be applied in WS devices. Although there has not yet been any demonstration of plasmon-enhanced WS using plasmon-light scattering, planar geometries will likely be chosen (Fig. 2d), as they have been under study for solar cells (Fig. 2c) that exploit the light trap [41, 42].

**Fig. 3:**
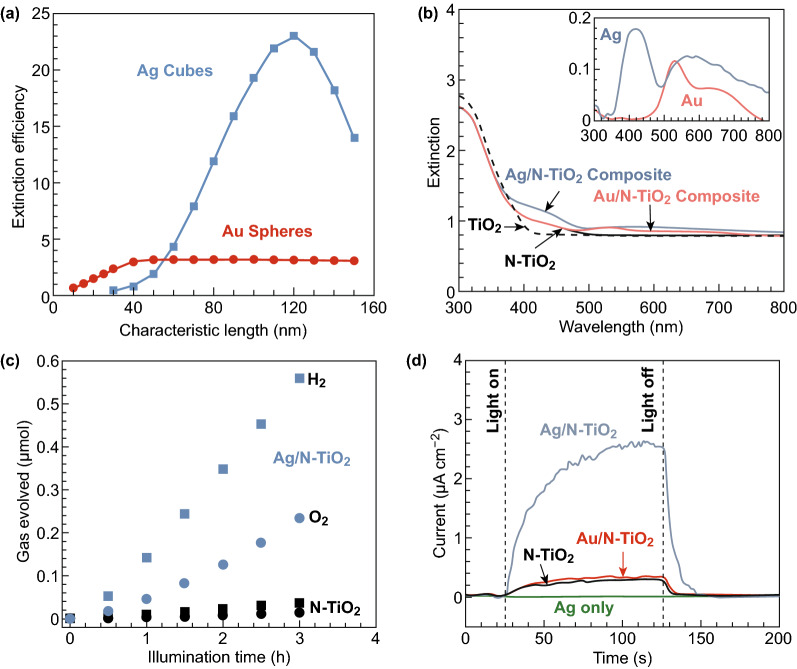
**a** Extinction efficiencies for Ag cubes and Au spheres as functions of particle size. **b** UV–Vis extinction spectra of samples. **c** H_2_ and O_2_ production upon visible illumination of photocatalysts. **d** Photocurrent responses upon illumination with a broadband visible light source. Reprinted with permission from Ref. **[**47**]**, Copyright © 2011, American Chemical Society

There are several reports available regarding the usage of plasmonic photocatalysts for WS. In 2011, considerable attention was given to the production of hydrogen by WS using a plasmonic photocatalyst under visible light illumination [43]. Thereafter, Ag film-based ZnO-coated nanorods of varying thickness on a polyethylene terephthalate flexible substrate photoanode were used for photoelectrochemical WS [44]. The fabricated photoanode of Ag nanoparticles in the (NPs)/WO_3_ film showed plateau photocurrent, which was about 1.6 times that for pristine WO_3_ photoanode without Ag NPs [45]. Further, Ag@SiO_2_@TiO_2_ core–shell photocatalysts with different thicknesses of SiO_2_ interlayer were fabricated, and significant photocatalytic activity was obtained by Zhang et al. [46] under visible light. Ingram et al. [47] reported WS on composite plasmonic-metal/semiconductor photoelectrodes using Au-NPs or Ag NPs onto nitrogen-doped TiO_2_ (N-TiO_2_). Although the reported conductivity and electronic structure of Au and Ag are similar, Au-surface plasmon resonance shifted relative to that of Ag and did not overlap expressively with the absorption spectrum of N-TiO_2_, as shown in Fig. 3. The overall WS suggested due to stoichiometric amounts of H_2_ and O_2_ was produced by photoelectrodes with a broadband visible source, as shown in Fig. 3c. The effect of Au-NPs is small on the photocurrent, but the addition of plasmonic Ag–NPs to N–TiO_2_ by ten times increases the visible light photocurrent as shown in Fig. 3d.

### Physics and Surface Chemistry for Optimization of WS

**Fig. 4:**
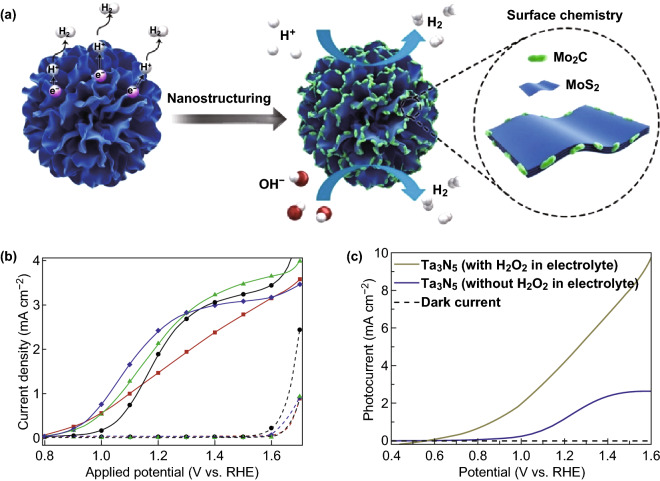
**a** Schematic represent of the MoS_2_/Mo_2_C; reprinted with permission from Ref. **[**50**]**, Copyright © 2019, Springer Nature. **b** Current densities of photoanodes in the dark (broken lines) and under simulated solar are shown as a function of the applied potential, *V*, with respect to the RHE. An atmospheric pressure chemical vapor deposition sample covered with one ALD cycle of Al_2_O_3_ has been measured after deposition (red squares), after annealing for 20 min at 300 °C (green triangles) and after annealing for 20 min at 400 °C (blue diamonds), sample before ALD (black circles). Reprinted from Ref. **[**53**]** with permission from The Royal Society of Chemistry, **c** *J*–*V* curves of Ta_3_N_5_ thin film photoanode in the electrolyte with and without H_2_O_2_ sacrificial agent. Reprinted from Ref. **[**54**]** with permission from the Centre National de la Recherche Scientifique (CNRS) and The Royal Society of Chemistry

The semiconductor photoelectrodes involve in complex chemical, physical, and electrical processes during the process of solar WS reaction, thereby operating the photogenerated electron–hole pairs for redox reactions [48]. Recent WS research has established three steps for the process: charge carrier generation in the semiconductor photoelectrodes, migration of charge carriers from the bulk to the surface, and redox reactions on the surface reaction sites. The activities of the photoelectrodes can be determined by the efficiency of these steps. Subbaraman et al. [49] enhanced the hydrogen evolution activity in WS by tailoring Li^+^–Ni(OH)_2_–Pt interfaces. They found that controlled arrangement of Ni(OH)_2_ nanoclusters on the surface of platinum electrode will enhance the activity of catalyzing the hydrogen evolution reaction by eight times compared to metal oxide catalysts. Recently, Luo et al. [50] reported surface chemistry engineering toward universal-pH catalysts for hydrogen evolution. On the basis of these studies, an efficient catalyst for hydrogen evolution reaction at high current density over a range of pH values was manufactured. Microspheres are composed of radially aligned MoS_2_ nanosheets with Mo_2_C nanoparticles at their edges (Fig. 4a).

The surface engineering approaches have been extensively used for developing the photoelectrochemical performance of nanostructured photoelectrodes. One of the most important methods to passivate the surface states of semiconductor photoelectrodes is atomic layer deposition (ALD). This technique is used to produce thin films with exact controlled thickness and proper growth over complex nanostructures [51]. This ideal modern method controls the surface states without intensely changing the surface properties or eliminating the injection of charge carriers [52]. Le Formal et al. [53] claimed that an ultrathin coating of ALD-grown Al_2_O_3_ can decrease the overpotential of *α*-Fe_2_O_3_ photoanode and, subsequently, enhance the photocurrent density (Fig. 4b). Zhang et al. [54] showed that the addition of a sacrificing agent such as H_2_O_2_ into the electrolyte enhances the photocurrent of the Ta_3_N_5_ thin film photoanode (Fig. 4b).

### Electrode–Electrolyte Interface Engineering Approach for WS

Water oxidation and reduction take place at the electrode–electrolyte interfaces; therefore, extremely large surface area of photoelectrodes is important for enhancing its efficiency in WS. The advanced surface engineering that involves deposition of mono- or multilayer modifiers at the semiconductor–electrolyte interface has a crucial role in improving photoelectrochemical performances. After surface modification, several new interfaces appear, such as the semiconductor–modifier interface, the modifier–electrolyte interface, and additional interfaces between adjacent layers of modifiers in the case of multilayer modification. Proper interface engineering of the active semiconductor electrode–electrolyte contact is the main challenge of a water splitting device. The crucial part is to form semiconductor–passivation layer co-catalyst–electrolyte interfaces, which would be capable of achieving the desired reactions without potential and current loss. Since the early stage of photoelectrochemical WS development, the solid–liquid interface has attracted much attention. Currently, several reports are available regarding the tuning of the efficiency of solar WS using electrode–electrolyte interface engineering. Zhang et al. [55] reported interface engineering of monolayer MoS_2_/GaN hybrid heterostructure for photocatalytic WS application. Kang et al. [56] summarized the details regarding the interface engineering of modulation charge carrier nature in ZnO photoelectrochemical WS.

### Defect Engineering for WS

The concept of defect engineering is correlated with the reconstruction of a surface to reduce the density of surface defects. The approach of defect engineering is to tune the electronic structure to improve photoelectrochemical performance by the formation of a surface disorder or intrinsic defects. Recently, a study has confirmed that defect engineering is an effective approach to modulate the electronic band structure, charge carrier transfer, and surface-active sites construction of photocatalysts [57]. The defect in semiconductors is of four types depending on the dimensions of the crystal lattice; they are 0D point defects, 1D line defects, 2D planar defects, and 3D volume defects, respectively [58]. 0D point defect controls the nonstoichiometric compositions that are related to the conductivity of most semiconductor materials.

**Fig. 5:**
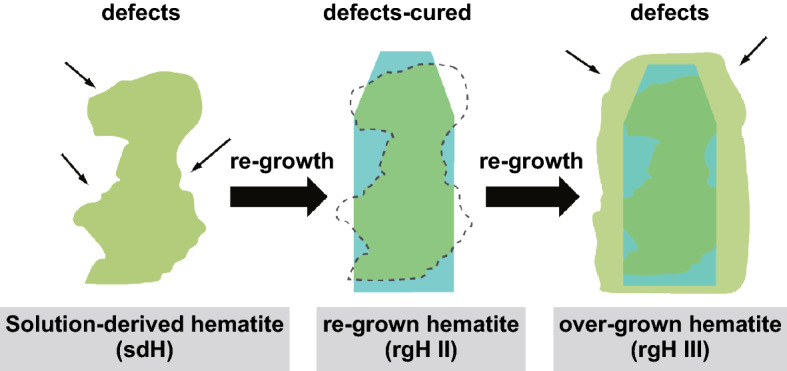
Defect engineering using re-growth scheme of hematite. Reprinted with permission from Ref. **[**69], Copyright © 2015, Springer Nature

Several methods have been introduced to deal with surface defects in semiconductors: hydrogen gas annealing [59], thermal annealing [60], flame annealing [61], partial oxidation [62], aluminothermic reduction [63], ultrasonication [64], chemical reduction [65], and electrochemical treatment [66]. In 2011, the concept of creating surface disorder was first demonstrated on TiO_2_ photoelectrode for photoelectrochemical WS [59]. Further, the research claimed that the control of intrinsic surface defects (oxygen vacancies) can expressively lead to the growth of photoelectrochemical performance of metal oxide photoelectrodes [67]. Cho et al. [68] claimed surface oxygen vacancies on different metal oxides by introducing a general flame reduction method. Figure 5 represents the solution-based re-growth procedure to study the hypothesis, wherein pre-formed hematite is exposed to acidic solutions under which condition dissolution (of Fe_2_O_3_) and deposition (of FeOOH) occur concurrently [69].

### Biological Approach for WS

Artificial photosynthesis systems store solar energy and reduce CO_2_ chemically. Photosynthetic reactions are mainly determined by three stages of reaction processes: the photosynthetic process; charge generation and separation processes; and catalytic chemical reaction processes. The overall efficiency depends on the kinetics of these processes and the balance of thermodynamics. In recent decades, in-depth studies/experiments have focused on further investigation of the mechanisms involved in natural photosynthesis. In particular, the structure of the O_2_-evolving site has been revealed in photosystem II by researchers recently [70], thus giving new inspiration for the design of artificial photosynthetic structures.

WS is seen as an environmentally friendly and one of the most exciting ways to produce renewable fuels with abundant resources using solar power and photocatalytic semiconductors. Since the discovery of this method, hundreds of diverse semiconductors have been developed and tested for half the reactions (either H_2_ or O_2_ production) that involve WS, but are expensive [17]. Cost-effectiveness is a crucial goal of the entire WS community. For industrial applications, a necessary, non-debatable fact is that the methods employed must be robust, inexpensive, and efficient. Developing a photocatalyst that meets these requirements for WS remains a serious challenge within the field.

**Fig. 6:**
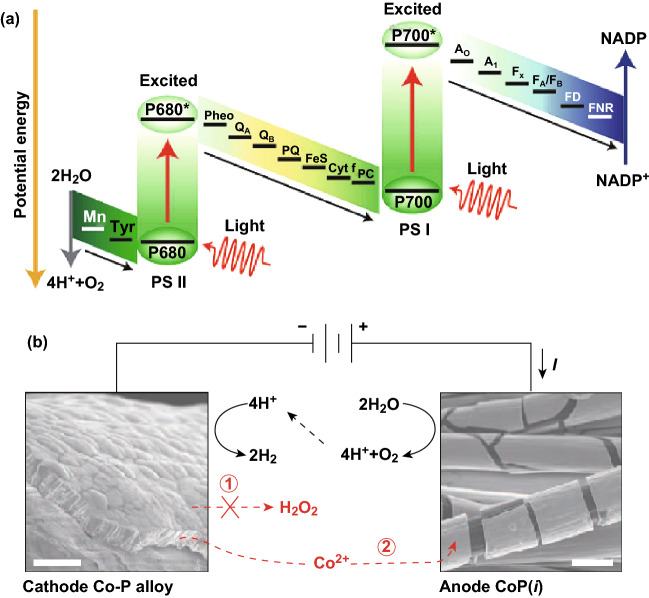
Active water splitting catalyst pair with minimal biological toxicity. **a** Natural photosynthesis pathway and **b** reaction diagram and scanning electron microscopy images for Co–P alloy cathode and CoPi anode. Reprinted with permission from Ref. **[**9**]**, Copyright © 2012, Springer Nature and Ref. **[**75**]**, Copyright © 2016, American Association for the Advancement of Science

Nature splits O_2_ and H_2_ equivalent species into water by a dual excitation process, with the two half-reactions separated in PSII and PSI as shown in Fig. 6a [71]. Inspired by natural photosynthesis, Byrd proposed a conforming system composed of two inorganic semiconductor photocatalysts in 1979. This has been further developed based on recently developed photocatalysts [72, 73] on RH-doped SrTiO_3_ or Ta_2_ as H_2_, but there has been limited success. Tachibana et al. [9] summarized some of the recent evolution in the development of artificial photosynthesis devices, with their subtypes for organic photosynthesis (biological photosynthesis), thereby focusing on the development of visible light-activated heteronanostructures including techniques that require an underlying interfacial carrier dynamics.

Martin et al. [74] demonstrated that strong organic semiconductors such as graphitic carbon nitride can be integrated into natural photosynthesis in a nature-induced WS system corresponding to PSII and PSI. They developed two parallel systems for complete WS under visible light incorporating two different metal (BiVO_4_ and WO_3_) oxides with graphitic carbon nitride. The facilitator is very important here because it significantly inhibits rapid repulsive recombination of charge, which is consistent with the electron chain transport between PSII and PSI. Given such benefits of a double excitation process, there are many researchers working on either two photocatalysts each favoring H_2_ or O_2_ production or a new mediator, which allows the charge between two photocatalysts to move efficiently.

More recently, Liu et al. [75] established a biological hybrid inorganic system that uses artificial leaf catalysts in blend with a bacterium *Ralstonia eutropha* to drive an artificial photosynthesis process for biomass and carbon fixation in liquid gels. In order to improve a biocompatible catalytic system that is not toxic to bacteria and is highly useful to WS, they introduced a reactive oxygen species (Fig. 6b, Route 1), wherein cobalt–phosphorus (Co–P) resistant alloy cathode is used. This alloy conducts the H_2_ growth reaction, while the self-healing CoPi anode conducts the O_2_ growth reaction [76]. The electrode pair serves to maintain dissipated cobalt ions in low concentrations and to give a low applied voltage that splits water to generate H_2_ for *Ralstonia eutropha*, which results in CO_2_ in organic complex molecules at high-efficiency support shortages.

### Modeling Approach for WS

Solar-to-H_2_ conversion efficiency, an important metric for evaluating the enactment of photoelectrochemical WS devices, is defined as a quantity of chemical energy produced as H_2_ is divided by input solar energy, with no externally applied bias. This is the most essential efficiency metric for comparing material and device performance and is useful in research for wide-scale benchmarking and comparison [77]. Modeling the theoretical device efficiency for photoelectrochemical WS is important as it can identify performance targets, guide the materials development process, and highlight aspects of device performance that need improvement. Researchers such as Weber and Dignam [78], Bolton et al. [79], and Rocheleau and Miller [80] expanded these initial analyses to photoelectrochemical WS to establish fundamental limits in that field.

The stability of the photoelectrodes against oxidation is regulated by the relative energy between their valence band maximum and intrinsic oxidation potential [81]. Such a complex interplay results in a multi-property optimization problem that is difficult to solve experimentally, especially due to challenges related to the structural characterization of interfaces. Theoretical models and computational studies therefore provide powerful tools to investigate photoelectronic interfacial properties and complement experiments. In particular, with recent advances in high-performance computing and sophisticated electronic structure theories and codes, the ability for routine modeling and simulation as a predictive tool is increasing rapidly, and now, it is a first-principles approach to electronic structure computation. The base approach may use molecular dynamics to accelerate material discovery for photoelectronic applications [82]. For example, it was demonstrated that first-principles calculations can be worked to scan combinations of 1000 elements throughout the periodic table to suggest new photoelectrode candidates [83].

**Fig. 7:**
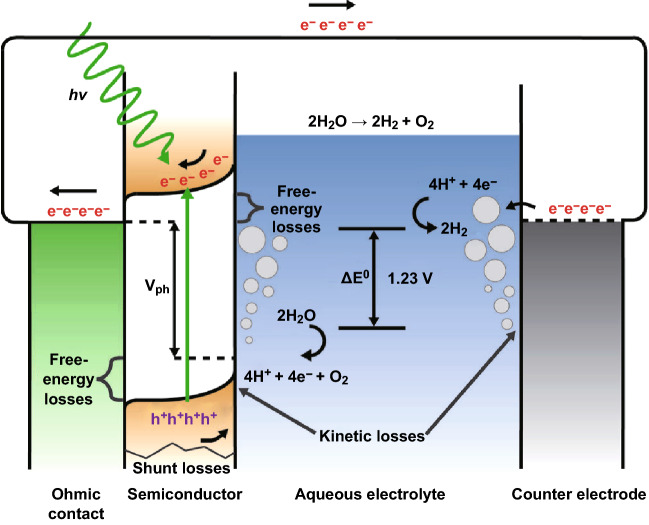
Schematic representation of energy diagram of a photoelectrochemical cell for the photoelectrolysis of water with various losses. Reprinted with permission from Ref. **[**86**]**, Copyright © 2014 WILEY-VCH Verlag GmbH & Co. KGaA, Weinheim

In photoelectronic solar fuel conversion, an intermediate (medium) is converted into fuel with the benefit of sunlight and photoelectrochemical active materials. In the simplest way, it is the production of H_2_ by splitting water. Figure 7 illustrates the principle of a photoelectrochemical cell operation for WS in an alkaline environment. The three major disadvantages are related to the free energy losses in the photoexcited state. Electrons are excited from their ground energy state (i.e., valence band) to the excited state (conduction band) with the assistance of light illumination, which leaves a positively charged hole in the valence band. Consequently, the formation of an electron–hole pair is achieved.

In an n-type semiconductor, electrons travel to counter the electrode, on which they deplete water and form H_2_ gas (4H_2_O + 4e^−^ → 4OH^−^ + 2H_2_). Holes move to the surface, at which they oxidize water to form oxygen gas (4OH^−^ + 4h^+^ → 2H_2_O + O_2_) [83]. Charge transport is restricted by the nature of semiconductor; electrode reactions are limited by the catalytic activity of the electrode material. Thus, both semiconductor physics and surface chemistry have to be carefully considered for optimization of photoelectrochemical systems [84, 85]. Photoelectrode materials suitable for efficient solar H_2_ generation must meet the following requirements:

1. Strong (visible) light absorption.
2. High chemical stability in the dark and under illumination.
3. Efficient charge transport in the semiconductor.
4. Appropriate band-edge positions to enable reduction/oxidation of water.
5. Low overpotentials for the reduction/oxidation reactions.
6. Low cost.

Open challenges in first-principles predictions of processes relevant to solar-to-fuel conversion include simulations of interfaces not only with water but also with aqueous solutions under various pH conditions. Although some attempts have been made to inspect the interaction of solvated ions in contact with TiO_2_ surfaces, and ion effects on the electronic properties of the electrode [87, 88], these types of study are rather sparse due to the limitation in time and length scale accessible by first-principles approaches. Coupling first-principles molecular dynamics to advanced sampling methods [89] may help alleviate this problem in the near future. In addition, the use of techniques such as effective screening medium methods [90] may be suitable to study ion effects at photoelectrochemical interfaces during operation. Finally, electronic properties of ions at interfaces need to be better understood, and this is an area of active research [91] with some recent progress obtained by combining advanced hybrid functional with GW calculations [92].

Another open theoretical challenge is the description of charge transport properties, such as charge mobilities, which ultimately determine the device’s efficiency. Evaluations of hole (p) and electron (e) mobilities as present in the literature, even if carried out from first principles, are still based on approximate theories that are often capable of yielding only correct orders of magnitude [93]. A unified first-principles approach that can describe the hopping (polaron) and band transport on the same footing is not yet available, and its development and application to realistic systems are a major challenge in predicting device efficiency for solar-to-fuel productions. In particular, although some recent progress was made on the study of the electron transfer rates at oxide interfaces [94], these calculations were based on transition-state theory and Fermi’s golden rule, which usually focuses on a single process at a time.

### Thermodynamic Approach

The solar thermochemical route additionally guarantees to be a suitable strategy for achieving this objective. The artificial chemical process and photovoltaic-powered electrolysis of water are promising approaches, though their implementation is somewhat restricted due to its low solar-to-fuel conversion efficiency (*η*_solar-to-fuel_) of < 5% and < 15%, respectively [95]. The alternative strategy is the solar thermochemical method that gives theoretically a high efficiency and allows large-scale production of H_2_ by using the entire solar spectrum [96]. One potential way to turn out H_2_ and carbon monoxide gas is to use thermochemical solar-driven cycles for WS. The obtained gases will be either used as a fuel directly or processed in a Fischer–Tropsch plant to get artificial hydrocarbons. Several thermochemical cycles have been investigated and evaluated within the past few years [97]. The cycle that has been subjected to the most analysis is the ballroom dancing cycle [98]:

1 $$\begin{aligned} & {\text{Endothermic}} - {\text{metal}}\;{\text{oxide}}\;\left( {\text{MO}} \right)\;{\text{reduction:}} \\ & {\text{MO}}_{x} \to {\text{MO}}_{x - \delta } + 0.5\delta {\text{O}}_{2} \\ \end{aligned}$$

2 $$\begin{aligned} & {\text{Splitting}}\;{\text{step:}} \\ & {\text{MO}}_{x - \delta } + {\text{H}}_{2} {\text{O}} \to {\text{MO}}_{x} + \delta {\text{H}}_{2} \\ \end{aligned}$$

**Fig. 8:**
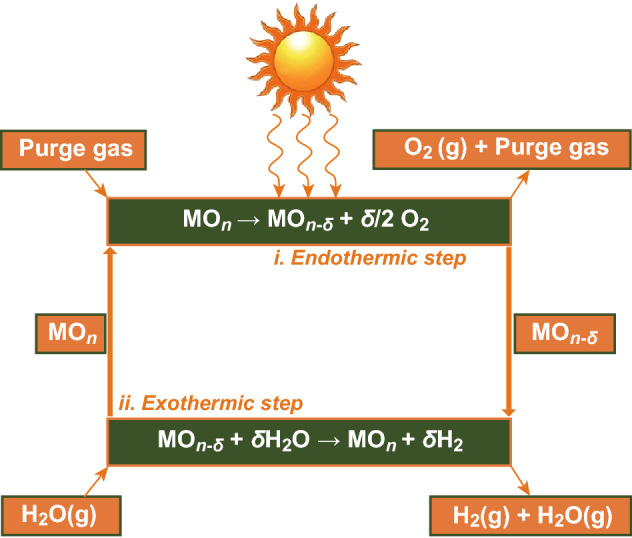
Schematic representation of the two-step solar thermochemical splitting of H_2_O using nonstoichiometric metal oxide redox pairs and concentrated solar energy **[**100**]**

The two-stage metal oxide process performed with the support of solar concentrators removes/eliminates the necessity to separate H_2_ and O_2_. The metal oxide reduces the metal or goes to a less valent metal oxide with the O_2_ (g) release during the endothermic (*T*_red_) step, and in the next step, it re-oxidizes the reaction (*T*_oxd_) with release of a stoichiometric amount of H_2_ (g), as shown in Fig. 8. In the two-step process, T_red_ > T_oxd_ is the thermodynamic driving force to become viable as described by Eq. 3 in terms of the free energy (Δ*G*) change in the formation of H_2_O ($\Delta G_{{f,T_{\text{oxd}} }}^{{{\text{H}}_{2} {\text{O}}}}$) and the entropy of O_2_ ($S_{{T_{\text{red}} }}^{{{\text{O}}_{2} }}$). Increasing *T*_red_ temperature increases entropy $S_{{T_{\text{red}} }}^{{{\text{O}}_{2} }}$, so the decrease in Δ*T* window can be permitted at higher *T*_red_. Conversely, at lower *T*_red_, Δ*T* has to be higher:

3 $$\Delta T = T_{\text{red}} - T_{oxd} = - 2\Delta G_{{f,T_{\text{oxd}} }}^{{{\text{H}}_{2} {\text{O}}}} /S_{{T_{\text{red}} }}^{{{\text{O}}_{2} }}$$

Thermal reduction of the oxide and WS have been conducted at the same temperature (*T*_red_ = *T*_oxd_ = *T*_iso_) with a large pressure swing acting as the driving force in the gas composition between the reduction and oxidation processes [99, 100].

Using solar thermal channel, WS significantly depends on the accessibility of suitable solar reactors. The construction of a solar reactor capable of operating a variety of cycles, materials, and temperature systems has been discussed in some reviews in detail [101, 102]. The solar reactors have been applied to nonstoichiometric oxide-driven, nonvolatile, and twisted two-step cycles. The solar configuration consists of three fundamental parts: concentrator, receiver, and reactor. Ferrite has been widely investigated using solar radiation, and there have been some attempts to use CeO_2_ in solar reactors. Perovskite oxides have not been employed so far in solar reactors for two-step WS purposes.

**Fig. 9:**
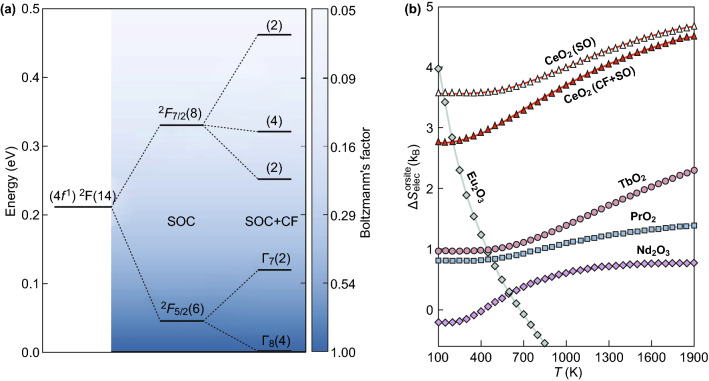
**a** Energy levels of the 4f one orbital of Ce^3+^. Ce^3+^ splits initially by spin orbit coupling and subsequently by cubic crystal field of the fluorite structure. **b** Calculated $S_{\text{elec}}^{\text{onsite}}$ for lanthanides ions. Reprinted with permission from Ref. [103**]**, Copyright © 2017, Springer Nature

Recently, Nagahvi et al. [103] reported an interesting thermodynamic approach based on large solid-state entropy reduction, which can increase thermodynamic efficiency, such as that of metal oxides, with different entropy for two-phase thermochemical WS cycles and onsite electronic configuration entropy, which are generated by coupling orbital momentum (*l*) and spin angular momentum (*s*) in lanthanide f orbitals. Figure 9a shows the *f*^1^(Ce^3+^) energy-level splitting that occurs during spin–orbit coupling, with calculated crystal field. Without crystal field, the *f*^1^ states split into ^2^F_5/2_ and ^2^F_7/2_ separated by using approximately 0.28 eV. The crystal field interaction further splits the sixfold degenerate ^2^F_5/2_ ground state into a fourfold degenerate Γ_8_ and twofold degenerate Γ_7_ subsets, separated by 0.12 eV. Calculated crystal field by opposing crystal potential method for Ce^3+^ splits the eightfold ^2^F^7/2^ degenerate energy states with energies 0.25, 0.32, and 0.46 eV.

Myers and Graves [104] took out the electronic entropy involvement from absolute entropy data of lanthanide (Ln^3+^) ions in lanthanide trihalides, which are comparable with the new approach of Naghavi et al. [103]. Having the energy $E_{i}$ and degeneracy ($g_{i}$) of each microstate, $S_{\text{elec}}^{\text{onsite}}$ of a system with m different microstates was calculated by the following equation, where $p_{i}$ is the probability of thermal excitation and *Z* is the partition function.

4 $$S_{\text{elec}}^{\text{onsite}} = - k_{\text{B}} \mathop \sum \limits_{i}^{m} g_{i} p_{i} \ln p_{i}$$

5 $$p_{i} = \frac{{\exp \left( { - E_{i} /k_{\text{B}} T} \right)}}{Z}$$

6 $$Z = \mathop \sum \limits_{i}^{m} g_{i} \exp \left( { - E_{i} /k_{\text{B}} T} \right)$$

For thermochemical WS cycle applications, the electronic absolute entropy does not matter; the entropy difference only before (*f*^*n*^) and after (*f*^*n*+1^) reduction is relevant $S_{\text{elec}}^{\text{onsite}} = 2(S_{\text{elec}}^{n - 1} - S_{\text{elec}}^{n} )$. The factor two is due to the circumstance that two Ce^4+^ ions are reduced due to oxygen vacancy because of the large $S_{\text{elec}}^{\text{onsite}}$ gain in occupying one of an *f*-orbital. The electronic entropy of reduction in the two-step thermochemical WS cycle systems reaches its maximum, that is, the entropy of oxidized state is zero,$S_{\text{elec}}^{n} = 0$. Using these criteria, Naghavi et al. [103] found largest $S_{\text{elec}}^{\text{onsite}}$ in Ce^4+^→ Ce^3+^, which undergoes an *f*^0^→ *f*^1^ redox reaction as shown in Fig. 9b. Having the oxidized state *f*^0^ (1S) with zero onsite electronic entropy is a unique feature of ceria compared to any rare earth ration. The largest second value was found in terbium (Tb^4+^→Tb^3+^) because non-subtracted Tb^4+^ has half-filled shell levels with only eight spin-degenerate states (2S + 1), but only one orbital (*L* = 0) degeneracy, while Tb^3+^ has an orbital degeneracy of 7 (*L* = 3), which provides additional entropy. This additional source of entropy may make Tb^4+^-based materials promising candidates for thermochemical WS cycle applications, since Tb, like Ce, is stable in two valence states (Tb^4+^/Tb^3+^).

### Solar WS by Enhancing Charge Separation Approach

Photoelectronic WS is an attractive technique for producing H_2_ and O_2_ from water without causing pollution to the environment. Efficient and low-cost transformation of solar energy into a transportable storable form is an essential goal, and photoelectrochemical splitting of water into gas H_2_ and O_2_ is a promising solution. Solar photons ${\text{h}}\vartheta$ with more than 1.23 eV should be sufficient, depending on the free energy required to split the water. However, the required energy is significantly higher as a product of kinetic and thermodynamic non-ideals in the actual oxidation (occurring at the anode) and reduction (occurring at the cathode) reactions. Monoclinic clinobisvanite bismuth scheelite (ms-BiVO_4_) has recently attracted a lot of attention because of its beneficial properties such as suitable conduction band-edge position, ability to withstand oxidation conditions, and material abundance [105] for on-demand oxygen production.

In addition, heterogeneous catalysts for the enlargement of oxygen are dominated by state-of-the-art metals, which dominate the noble metals (Ir or Ru), but their scarcity and high price have significant technological implications. Abundant and inexpensive first-row transition metal oxides are also water oxidation competent catalysts, although exclusively at very high pH, or with auxiliary or ancillary electrolytes (i.e., phosphates) [106]. Alternatively, the Prussian blue-type material has emerged as a favorable catalyst for water oxidation catalysis [107, 108]. These coordination polymers are: easy to process and obtain by using soft chemistry methods, available as nanoparticles/thin films, stable and active in a very large pH range, from neutral down to extremely acid conditions, and are non-toxic.

The unique performance of these catalysts is based on their structural and electronic characteristics. They are constructed from a hexasanomatolate anionic complex and a metal di-cation in stoichiometric addition. These coordination networks are very strong due to the strength of cyanide bridging, but with a rather high covalent character. Their structure is modeled after a face-centered cubic ideal network, but their nonstoichiometric nature creates many voids for solvent (water) and counter cations.

Recently, the ternary oxide BiVO_4_ topped all metal oxide photoanodes owing to a relatively small (2.4 eV) band gap, which provided efficient light absorption, had reasonably negative conduction band edge (~ 0 V vs. reversible H_2_ electrode), and allowed moderate charge transport properties [109]. Theoretically, the maximum water oxidation photocurrent (*J*_max_) for BiVO_4_ photoanodes under 1.5 global air mass solar illumination is 7.5 mA cm^−2^ [109]. According to $J\left( {{\text{H}}_{2} {\text{O}}} \right)$, the practical water oxidation photocurrent ($J\left( {{\text{H}}_{2} {\text{O}}} \right)$) is considerably lower owing to the limited light absorption $\left( {\eta_{\text{abs}} } \right)$, charge separation $\left( {\eta_{\text{sep}} } \right)$, and surface charge transfer $\left( {\eta_{\text{trans}} } \right)$ efficiencies of the BiVO_4_ material; nevertheless = *J*_max_ × $\eta_{\text{abs}} \times \eta_{\text{sep}} \times \eta_{\text{trans}}$ [33].

Various efforts were made to increase these capabilities. In particular, charge transfer efficiency $\eta_{\text{trans}}$ has been dramatically improved on the BiVO_4_ surface to improve water oxidation kinetics and/or surface malfunction by coating the oxygen oxidation reaction catalyst [110, 111]. The charge separation efficiency $\eta_{\text{sep}}$ of BiVO_4_ has been improved in several ways, including the introduction of nanoscale porosity [110, 112], reduction of BiVO_4_ thickness [111], introduction of electron donors such as Mo and W [110, 113], and formation of a distributed homojunction by the starter of a gradient doping concentration of W in a BiVO_4_ film [114]. In addition, ηsep has been shown to improve the formation of heterogeneities of BiVO_4_ with other materials including films (SnO_2_, SiO_2_, WO_3_, graphene) and nanowires (WO_3_ and Fe_2_O_3_) [114].

**Fig. 10:**
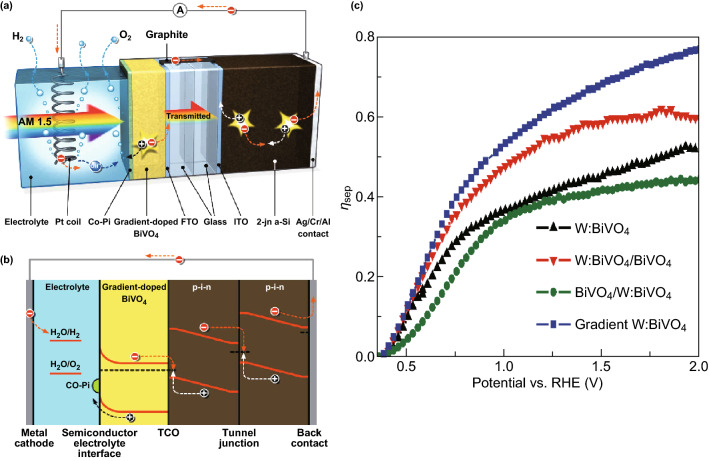
**a** Schematic diagram of the combined device of gradient-doped W/BiVO_4_ and a-Si solar cell. **b** The corresponding band diagram of the hybrid photoelectrode device. *ITO* tin-doped indium oxide, *TCO* transparent conducting oxide. **c** Carrier-separation efficiency ($\eta_{\text{sep}}$) as a function of applied potential for 1% W-doped BiVO_4_ (black triangle), W/BiVO_4_ homojunction (red inverted triangle), W/BiVO_4_ reverse homojunction (green circle), and gradient-doped W:BiVO_4_ (blue square). Reprinted with permission from Ref. **[**114**]**, Copyright © 2013, Springer Nature

Among the latest state-of-the-art BiVO_4_-based photoanoids, the highest efficiency was achieved by a doped BiVO_4_ film with gradient doping by W and an inherent SnO_2_ heterogenation, synthesized over a textured substrate, and cover coated with cobalt phosphate (CoPi). Oxygen evolution reaction catalysts have $\eta_{\text{abs}} , \eta_{\text{sep}} ,$ and $\eta_{\text{trans}}$, which were reported to have reversible water oxidation efficiencies of 1.23 V versus reversible H_2_ electrodes as 75, 60, and about 100%, respectively [114]. Figure 10 shows the higher values of till date carrier-separation efficiency of the W/BiVO_4_ homojunction reported by Abdi et al. [114], who reported that the use of dopant concentration differences is actually used to bend additional bands and thus to create/improve carrier separation. To fabricate a complete device for solar WS, which does not rely on externally applied bias potentials, they combined the state-of-the-art Co–Pi–W/BiVO_4_ photoanode with a two-junction a-Si superstrate photovoltaic cell located behind the photoelectrochemical cell, as shown in Fig. 10a, b [114].

### Nanomaterials Designing Approach for WS

Insulation and semiconductor nanostructures provide innumerable opportunities for manipulating/controlling the light at nanoscale. The higher their dielectric constant, the more strongly they interact with incident sunlight. When properly sized and shaped, they can also exhibit very strong optical resonances that can further promote light-to-light/light–matter interactions compared to bulk materials. It is significant to note that the strength of these resonances is similar to that found in metallic nanoparticles [115]. They can also occur in deep-subwavelength structures (~ 10 nm) and have already enabled the performance improvement of nanoscale optoelectronic devices that can integrate with same size semiconductor electronic components [116]. Recently, the photovoltaic community has begun engineering these resonances with the aim of improving solar cell performance. Excitation of these resonances in semiconductor nanostructures can directly enhance useful absorption if semiconductor nanostructures are a fundamental part of the dynamical solar cell material or indirectly by enabling excitation of guided and diffracted resonances. The way in which light is absorbed or dispersed is determined and directed by the nature of the localized optical resonance that can be excited in a nanostructure. In order to engineer the paramount possible antireflection coatings and light trap layers, it is thus important to classify different types of local resonances and gain an intuitive understanding of their behavior.

However, the demonstrated solar-to-H_2_ conversion efficiency of the material to date is low which is basically strained by its smaller carrier diffusion length. Most commonly, the actual width of the photoelectrode film is determined by the lifespan time of carrier transport. The power to use built architectures to deposit a denser thick coating layer of photoactive materials photoactive materials is important for superior photoelectrochemical WS and alternative electrical phenomenon systems [117, 118]. Nanocone structures are thought about in one in all the foremost promising candidates for high-efficiency skinny film electrical phenomenon [119]. However, few studies on nanocone-based photoelectrochemical devices have been explored, in particular with porous photoactive thin/thick layers on nanocone structures. In photoelectrochemical cells, photoactive coating layers deposited on a nanocone conductive substrate can not only improve the light absorption of photoactive materials, but also sustain efficient charge separation, provide a larger contact surface area, and promote surface water oxidation electrode/electrolyte interfaces.

**Fig. 11:**
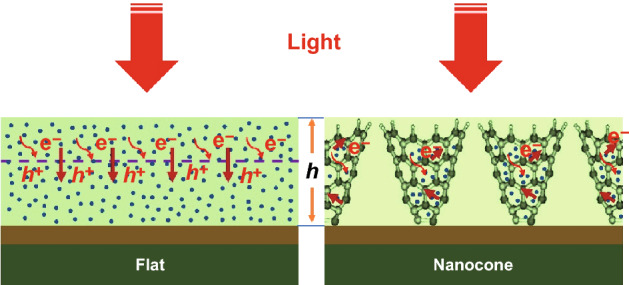
Schematic illustration of the optical absorption mechanism and electron transport of nanoporous BiVO_4_ on the flat substrate and the conductive nanocone substrate **[**21**]**

Qiu et al. [21] reported an unambiguous strategy for the depressions of nearly 700-nm-thick nanoporous Mo-doped BiVO_4_ (Mo/BiVO_4_) layer on engineered cone-shaped sub-nanostructures and demonstrated at low applied voltage that the exclusive photoanode attains a remarkable WS photocurrent with the best-reported solar-to-H_2_ conversion efficiency to date. The conductive nanocone substrate can accomplish efficient charge collection for the relatively thick film as shown in Fig. 11. In the aforementioned study, the authors introduced a thick coating of nanoporous photoabsorption layer through engineered cone-shaped nanostructures with an efficient higher charge separation, which resolved the immediate issue related to incompatibility of light absorption capacity with carrier transport length. The strategy of coating/depositing photoactive materials on engineered light-trapping architectures provides a technique for a new photoelectrode with high-performance photoelectrochemical WS cells [21].

Among the nanoparticle/nanotechnology-based electrodes used, WS-Fe_2_O_3_ (hematite) has occurred as one of the most promising materials [120, 121]. Hematite is attracting widespread interest for use in photoelectrolysis of water for H_2_ production because iron is one of the least expensive and most abundant metals and has a high theoretical solar-to-H_2_ conversion efficiency of 14–17% (based on the lower heating value of H_2_), which corresponds to a photocurrent of 11–14 mA cm^−2^ [122]. Despite improvements in its performance as high as 3 mA cm^−2^ under standard air mass and 1.5 illumination [123], structural features of hematite nanoparticle aggregates with the highest impact performance remain to be recognized by experimentation. In particular, although it has been projected that the reduction in electron–hole recombination in the synthesis of nanostructures with increasingly smaller dimensions will improve the photocurrent by decreasing electron–hole recombination [124], with nanostructure strategies, very little is acknowledged about the effect of such defects.

**Fig. 12:**
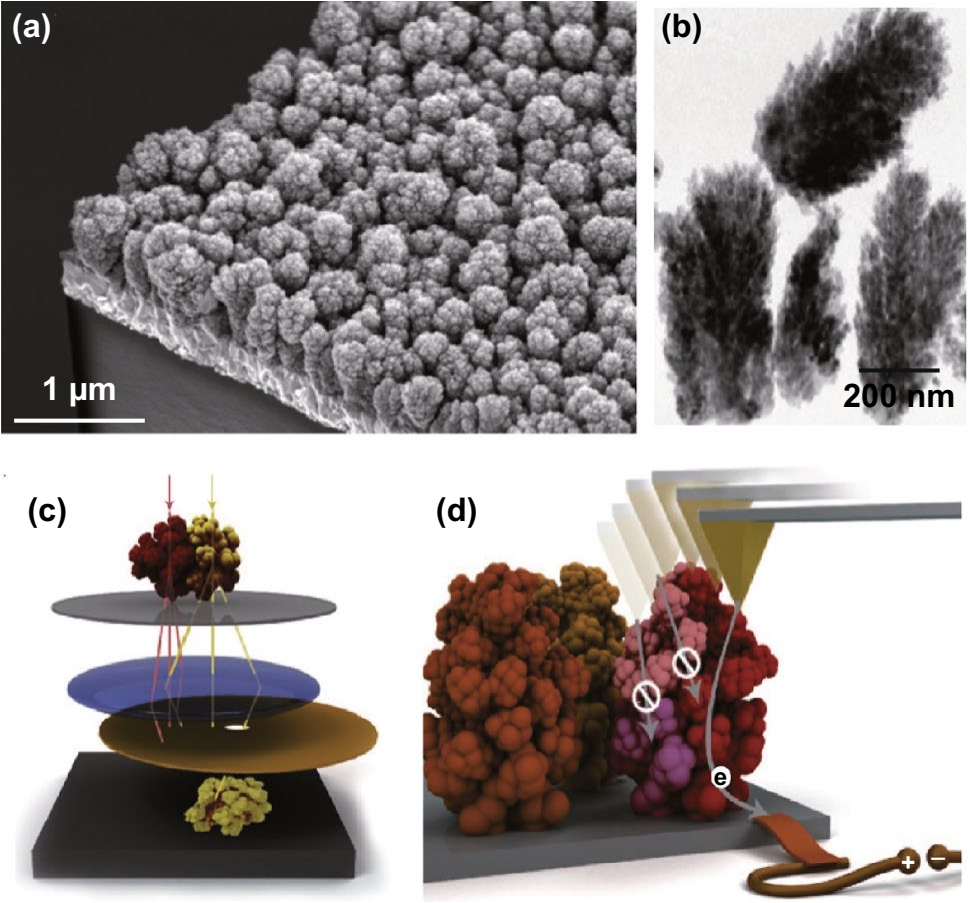
Classical structure analysis by SEM, BF-TEM, and identification of champion nanostructures. **a** Cross-sectional SEM images show electrodes from a 45 viewing angle. **b** The areal densities of nanoparticle aggregates. **c** A DF-TEM analysis allows each region of a nanoparticle aggregate with a unique crystallographic orientation to be imaged separately. **d** Analysis by C-AFM examines the charge transport properties of individual nanostructures. Each color represents a different crystal orientation. Reprinted with permission from Ref. **[**125**]**, Copyright © 2012, American Chemical Society

Recently, Warren et al. [125] developed an approach to correlate the spatial distribution of crystalline and current-carrying domains in whole nanoparticle aggregates and they called this approach as nanoparticle-based Fe_2_O_3_ applied to electrodes that are interested in solar-to-H_2_ energy conversion. Their studies suggested that champions are nanoparticle aggregates that are largely liable for the high photocurrent for their experimental samples. The structure reported using conventional characterization techniques, including electron microscopy (Fig. 12a) and bright-field transmission electron canopy (Fig. 12b), is shown in Fig. 12 [125]. The dark-field transmission electron microscopy method was based on crystal-by-crystal imaging of entire nanocrystal aggregates, which provided information about the spatial distribution of high- and low-angle grain boundaries (Fig. 12c). Using atomic force microscopy, they measured the carrier charge transport characteristics of the majority of these nanoparticle aggregates (Fig. 12d) [125].

These champion nanoparticle aggregates may have photon-to-current capabilities that reach theoretical limits. If the structural properties of these aggregates can be understood more fully, there is a possibility that the difference between the reported new benchmark and theoretical maximum efficiency can be bridged. When electrodes are invented with a high quantity of these defender nanostructures, the electrodes accomplish the highest photocurrent of any metal oxide photoanode for photoelectrochemical WS under 100 mW cm^−2^ air mass and 1.5 illumination.

### Artificial Leap Approach for Solar WS

To convert sunlight energy into chemical energy, the green leaf splits water through the photosynthetic natural process to produce molecular oxygen and H_2_, which form separate protons and electrons. The primary stages of natural photosynthesis include absorption of sunlight and its conversion into spatially distinct electron–hole pairs. The holes in this wire-free photocurrent are occupied by O_2_—evolving the complex of photosystem PSII to oxidize oxygen in the water. The proton and electron matrixes created as a by-product of a complex reaction that develops O_2_ are captured by ferredoxins of photosystem I. With the help of ferredoxin-nicotinamide adenine dinucleotide phosphate (NADP^+^) reductase, they are used to produce H_2_ in the form of nicotinamide adenine dinucleotide phosphate hydrogen (NADPH). To recognize the solar light energy conversion function of the leaf to produce a synthetic material, the light-absorbing material must capture/arrest a solar photon to generate/produce an electric current that is connected by the catalyst, which motivates the four electron/hole fuel-forming WS reaction under benign conditions and under 1 Sun (100 mW cm^−2^) illumination.

**Fig. 13:**
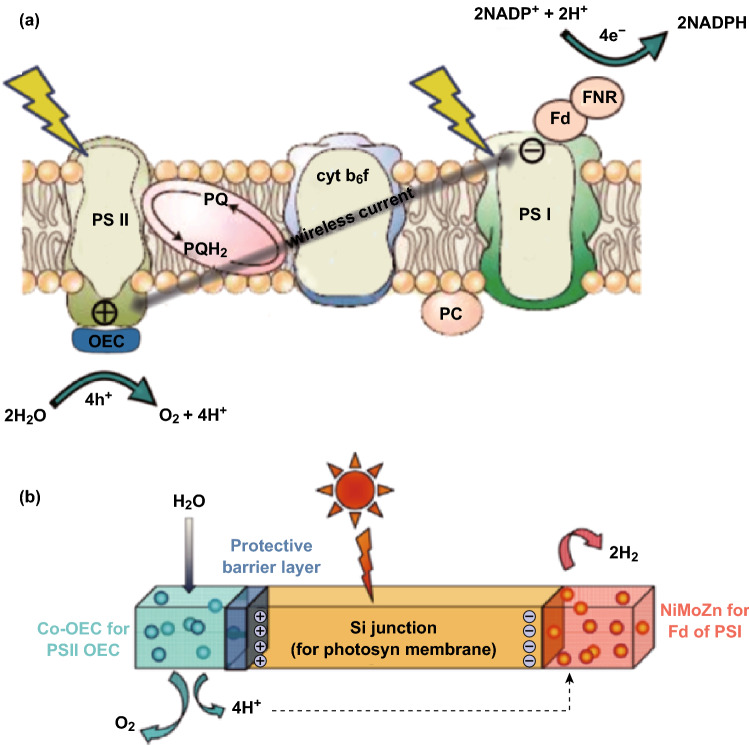
**a** A simplified scheme of the light-driven reactions of photosynthesis. **b** Construction of an artificial leaf **[**127**]**

The compound that receives WS in photosynthesis is the O_2_ evolving complex, which resides in photosystem II. The oxygen-evolving complex is ‘charged’ by a solar-driven wireless current as shown in Fig. 13a [127]. Light absorption at the reaction center of the light mechanism photosystem PSII produces an electronically excited electron. The hole (×4) resulting from electron transfer is passed within PS II to the oxygen-evolving complex, which performs the critical WS step to release O_2_ and four protons. The electrons are transferred/transported through a series of redox-active cofactors to photosystem I. These cofactors include plastoquinol and plastocyanin that bind within the cytochrome *b*_6_*f* (cyt *b*_6_*f*) complex, finally arriving at ferredoxin bound to photosystem I (Fig. 13 a). Through ferredoxin-NADP^+^ reductase (FNR), these energized active electrons reduce protons to produce H_2_ in the form of NADPH [126]. In implementing these reaction sequences, the plant stores solar energy in a fuel-forming process resulting from rearranging the bonds of water.

An artificial leaf containing earth-abundant materials which works under ordinary conditions and at 1 Sun illumination has been realized by interfacing NiMoZn and Co-OEC with a triple junction amorphous Si (3jn-a-Si) solar cell [27, 127].The ability to perform WS with Co-OEC and NiMoZn catalysts in natural waters, under ambient conditions, and at high and stable current densities provides a direct path to the creation of an artificial leaf, the design elements for which are shown in Fig. 13b [127]. The role of the photosynthetic membrane to capture solar light and convert it into a wireless current is assumed by Si. The photogenerated single electron and hole are relayed to the Co-OEC and NiMoZn catalysts, until the necessary four electron–hole equivalents are attained to drive the bond rearrangement of WS.

**Fig. 14:**
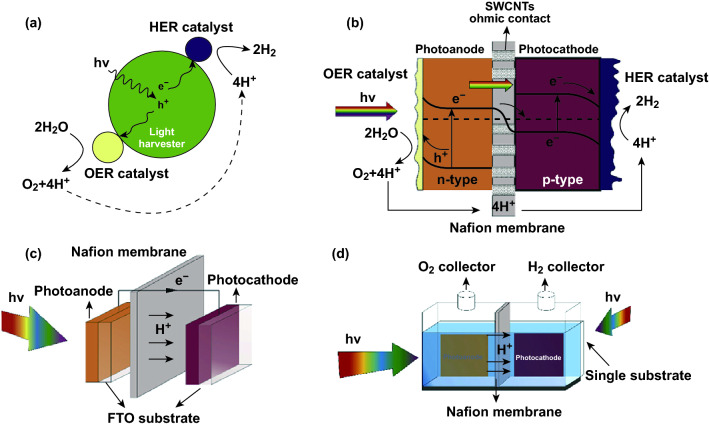
Design of an artificial leaf prepared of a single semiconductor having huge band gap (**a**), a dual photoelectrodes that are assembled with a proton exchange membrane in the back-to-back configuration (**b**), a dual photoelectrodes wired (**c**), and a dual photoelectrodes loaded on a single support in the side-by-side configuration (**d**). The dual photoelectrodes in the three latter cases are made of semiconductors with small or narrow band gaps. Reprinted with permission from Ref. **[**131**]**, Copyright 2017, Elsevier

Some semiconductors with a large band gap provide sufficient energy-driving force (e.g., *E*_g_ > 1.23 eV) as well as appropriate band-edge potentials to drive the overall WS process. Indeed, TiO_2_ (*E*_g_ ~ 3.2 eV) was the first material for WS applications [17]. Its conduction band-edge potential is −0.17 V in comparison with the normal H_2_ electrode (NHE), which is adequately reductive for the O_2_–H_2_ evolution reaction (HER), whereas valence band-edge potential is +3.03 V, with NHE being substantially sufficient for O_2_ evolution reaction (OER). The same condition is identified and recognized for graphitic carbon nitrile *g*-C_3_N_4_, TaON, CdS, GaN [128]. Thus, the simplest configuration of a non-natural leaf uses these wide band gap semiconductors as light harvesters. On the surface of these light harvesters, two appropriately suitable catalysts for the HER and OER are loaded [129, 130]. This configuration was developed mainly as nanoparticles suspended in solution (Fig. 14a) but also as a freestanding electrode system [131]. However, it is worth noting these large band gap semiconductors can only harvest UV and near UV photons that constitute about 5% of the whole solar spectrum. Thus, low solar to H_2_ conversion yield, being less of 1%, is usually expected.

Dual semiconductors having small or narrow band gaps were used to make artificial leaf. In fact, the natural photosynthesis coordination process chooses P680 and P700 chlorophyll, which exhibits a maximum absorption at wavelengths 680 and 700 nm as the dual light harvesters [130]. These are like an optical band gap of absorbent *E*_g_ of ca.1.8 eV. Inspired by this natural engineering, an artificial leaf is also proposed to be made up of two small or narrow band gap semiconductors (Fig. 14b). Photoanode is made of an n-type semiconductor, whereas p-type semiconductor was used to make the photocathode [132]. These two electrodes are wired/connected from side to side making ohmic contacts of high electrically conducting materials that have the appropriate work function level. Ideally, this contact material should exhibit a high transparency, so light-blocking phenomena are suppressed. Furthermore, when proton conductivity is disturbed, it is extremely demanding that ohmic contact materials should be implanted inside a proton exchange membrane such as Nafion^®^. Basically, CNTs embedded within a Nafion^®^ membrane were developed for a fuel cell of H_2_ proton exchange membrane [133]. Such membranes can be practical in principles, in artificial leaf engineering.

In fact, a complete artificial leaf design as described above (Fig. 14b) is still to be demonstrated. For instanced example, the photoanode and photocathode are loaded onto two different conducting substrates, for example, metal or translucent conducting oxides (TCO) such as fluorine (FTO)-doped or indium (ITO)-doped tin oxides. These electrodes are then wired on another ohmic wire contact such as metal (Fig. 14c). The inclusion of two photoelectrodes on a single substrate can easily be made as a side-by-side mode (Fig. 14d). A back-to-back assemblage, being similar to the design described and labeled in Fig. 14b, represents a major challenge [131]. For this, it is necessary that both these electrodes are grown one by one, in a step-wise manner. In other words, photoanode should be grown under experimental conditions those are compatible for stability of the photocathode and vice-versa. In this framework, the advance development of a solution rolleroll process for photoelectrode fabrication, which employs ambient experimental conditions, may be a possible approach. To this end, learning from the creation of organic photovoltaic is a possibility. Indeed, with this methodology, eye-catching advances have been reached for the fabrication of organic material-based photoelectrochemical or photoelectrodes cells [134–136].

**Fig. 15:**
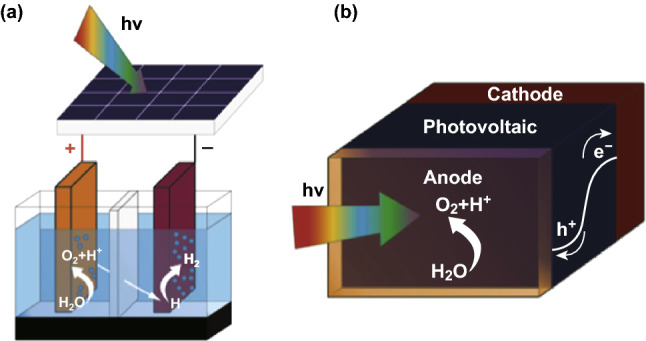
Artificial leafs made of photovoltaics e water electrolyser assemblage: **a** wired and **b** wireless. Reprinted with permission from Ref. **[**131**]**, Copyright 2017, Elsevier

An artificial leaf is designed through the simplest configuration and the dual combination of catalysts OER and H_2_ evolution HER reaction for photovoltaic and water electrolysis. Since the stability of a solar cell in water is indeed a major issue, this configuration allows photovoltaic cells to be excluded from contact with water (Fig. 15a) [131]. The structure of a wire-free artificial leaf is further challenging the construction. To this end, the catalysts HER and OER must form the buried junction with the photovoltaic material (Fig. 15b). Because the photovoltaic must be immersed in water for operation in this case, there are challenges to identify suitable strategies to stabilize the photovoltaic material. Indeed, Si photovoltaic is easily oxidized in water when soluble O_2_ concentrations exceed 15 ppb [137]. However, along with air humidity, emerging organic–inorganic perovskite photovoltaic material is encountered due to rapid decomposition in contact with water [138].

### Challenge of Charge Transport Properties for WS

The main target of this research is to produce high-efficient devices in the photoelectrochemical system, with low production cost and longer lifetime. In this context, designing a promising light absorber material that has favorable band alignment for water oxidation and possesses relatively long photocarrier lifetime is a mandatory requirement. This new material should not be restricted by poor carrier transport, deviations from ideal stoichiometry, and other defects. In particular, the selection of materials and approaches for future innovations is quite limited for surface protection, surface-state passivation, and selective charge extraction. New deposition approaches would be needed to fabricate promising materials that are semiconductor sensitive. For this purpose, extensive knowledge is required regarding interface energetics, the dynamic behaviors of interfaces during operation, and the electrolyte and temperature dependence of interface properties to design robust interfaces.

Although signification improvement has been made in this area in the past decade, constant developments are required for understanding surface and interface engineering properly to meet the demand for the progress of photoanodes with higher efficiencies in the practical application of photoelectrochemical WS. It is necessary to improve the understanding regarding the limitations of numerous surface modifications and how different parameters are involved in charge separation. The prediction of a right combination may be possible by understanding the transport mechanism of different semiconductors, thereby helping to reduce the risk that may arise because of ignoring potential materials and, in addition, helping to accelerate the progression of material screening. Finally, the problem related to charge transport could be solved by developing a strong theoretical base, in addition to knowledge regarding computational optimization and experimental skill. All of these would enable the designing of new materials for WS research.

## Conclusion and Future Prospects

Several combinations of semiconductor materials, electrocatalysts, and cell configurations are available for photoelectrolysis research. As solar fuel research expands, standardization of research methods and characterization techniques for accurate reporting becomes paramount and ultimately helps the field move into new areas of development and discovery. The use of a dual (D4) band gap water splitting cell configuration, as opposed to the use of a mono-band gap (S2) configuration for splitting of water, where the electric field is engendered at the semiconductor fluid junction or concluded a ‘buried junction,’ seems to be the furthermost effective and strong use of corresponding paired light-absorbing materials. Multiple junction configurations involve new contacts and new electrocatalysts. Semiconductor materials continue to develop and fully explain and optimize their many electrical properties. Recent efforts toward the development of capable photoelectrolysis equipment and advances help in controlling the size and shape of micro- and nanoscale features of semiconductors and catalysts. The use of structured photoelectrodes reduces material purity by controlling the directionality of charge movement and the light absorption pathways in semiconductors. High aspect ratio photoelectrode surfaces also might reduce the catalytic activities with the use of less expensive catalysts. The exploration for earth-abundant constituent materials that may be cast off in solar WS cells remains an essential target for inexpensive, globally, and ecofriendly benign approaches for energy conversion and storage. Stability of the photoelectrode remains to be a foremost challenge for the enlargement of competent photoanodes and photocathodes. Nature uses a regularly revived/renewed dual band gap photosystem to capture/arrest sunlight and store the energy in simple sugar molecules. A similar photoelectrochemical artificial strategy/approach can be recycled to decay water using two semiconductors and store/convert the energy in the simplest chemical bond H_2_.
